# Proteomics analysis of excretory-secretory proteins in mixed-sex adult stage *Toxocara canis*

**DOI:** 10.3389/fpara.2026.1965955

**Published:** 2026-10-27

**Authors:** Phawiya Suksomboon, Bruce A. Rosa, Kumar Sachin Singh, John Martin, Bin Zhan, Maria Elena Bottazzi, Makedonka Mitreva

**Affiliations:** 1 Department of Medicine, Washington University School of Medicine, St. Louis, MO, United States; 2 National School of Tropical Medicine, Department of Pediatric Tropical Medicine, Baylor College of Medicine, Houston, TX, United States; 3 Department of Genetics, Washington University School of Medicine, St. Louis, MO, United States; 4 McDonnell Genome Institute, Washington University in St. Louis, St. Louis, MO, United States

**Keywords:** excretory-secretory proteins, mass spectrometry, proteomics, *Toxocara canis*, toxocariasis

## Abstract

**Introduction:**

*Toxocara canis* is a prevalent zoonotic nematode responsible for toxocariasis in humans and animals. To better understand the molecular mechanisms underlying parasite persistence and host interaction, this study provides the first comprehensive proteomic characterization of excretory-secretory products (ESPs) from adult *T. canis*.

**Methods:**

ESPs were collected from mixed-sex adult worms and analyzed via LC-MS/MS.

**Results:**

A total of 468 proteins were identified. The secretome was dominated by highly abundant *Toxocara* excretory-secretory (TES-26) phosphatidylethanolamine-binding proteins, C-type lectins (Collectin-12), and superoxide dismutases (SOD), that accounted for 34 ESPs and 49.3% of total detected protein. Functional enrichment revealed a significant presence of classical secretion signal peptides, as well as proteins associated with the exosome pathway, indicating the utilization of non-classical secretion mechanisms. Comparative orthology analysis against the phylogenetically related (Clade III) nematodes *Ascaris suum* and more distant (Clade V) *Ancylostoma ceylanicum* demonstrated a strong enrichment for species-specific ESPs, as well as a subset of highly conserved ESPs across species.

**Discussion:**

These findings establish a foundational proteomic framework that enhances our understanding of *T. canis* biology and identifies promising targets for highly specific diagnostics and rational vaccine development.

## 1 Introduction

*Toxocara* spp., which includes *Toxocara canis* and *Toxocara cati*, is a significant zoonotic pathogen that poses a substantial public health risk worldwide in tropical and subtropical regions such as Europe, Asia, Africa, South America, and North America (5 to 15% seroprevalence) (Antonopoulos et al., 2024; Berrett et al., 2017; Sariego et al., 2012; Toochukwu Joy, 2017). Toxocariasis is primarily spread to people through raw vegetables or foods, water, sand, and soil which are contaminated with *Toxocara* spp. eggs, with 21% of soil samples collected worldwide being identified as contaminated (Fakhri et al., 2018; Rostami et al., 2020). The adult stage resides in the host intestine (including domestic animals such as dogs and cats) and excretes up to 200,000 eggs per day in their feces (Magnaval et al., 2001), facilitating transmission to humans. Humans become infected by accident by ingesting *T. canis* embryonated eggs shed in the feces of infected animals (Macpherson, 2013). Embryonated eggs hatch in the small intestine (Holland, 2023) and the larvae migrate through various tissues in humans while evading the immune system, including in the lungs, liver, eye, brain, and muscles, causing serious health conditions such as visceral larva migrans (VLM), ocular larva migrans (OLM) (Henke et al., 2023), and neurotoxocariasis which can lead to long-term damage if not diagnosed and treated early.

Globally, 1.4 billion people are estimated to be infected by or exposed to *Toxocara* (Rostami et al., 2019) resulting in an estimated 91,714 disability-adjusted life years (DALYs) and an annual estimated cost of $2.5 billion (Antonopoulos et al., 2024). Another study suggested that 3.9% to 84.6% of people in Southeast Asian countries and Taiwan (SEAT) are infected or exposed (based on seroprevalence and not confirmation of active infection) (Chou and Fan, 2020), and in Southern Thailand, *T. canis* infection prevalence was 58.2%, substantially higher than the global average of ~19% (Phasuk and Punsawad, 2020). However, human diagnosis relies on antigen-based detection, and existing antigen-based detection approaches suffer from cross-reactivity with antigens from other parasitic helminth infections, especially ascariasis (Muñoz-Guzmán et al., 2010), strongyloidiasis and other species (Cognato et al., 2020), so estimating exact worm burdens is difficult. Despite its importance, very little is known about the array of host-parasite molecular mechanisms that underlie the pathogenicity of *T. canis* and allow it to evade the immune system and survive in the host for years. Thus, identifying novel, highly specific candidate antigens is critical for improving immunodiagnostic methods such as Enzyme-Linked Immunosorbent Assay (ELISA) (Jin et al., 2013; Schaefer et al., 2022), Western blot (Morales et al., 2002), and Polymerase Chain Reaction (PCR) (Khademvatan et al., 2013; Winterfeld et al., 2024).

Excretory and secretory products (ESPs) are actively produced and released by helminth parasites, including proteins, proteases, lipids, glycoproteins, extracellular vesicles (EVs) (Wu et al., 2024), and other bioactive molecules (Lightowlers and Rickard, 1988). ESPs play critical roles in parasite survival, facilitating processes such as tissue penetration, immune evasion, nutrient absorption, modulating and evading host immunological responses (Harnett, 2014; Yeshi et al., 2022). In recent years, proteomics has emerged as a valuable technique for studying parasite secretome and identifying their intricate interactions with the host and its immune system (Dabrowska et al., 2023; Qiu et al., 2024; Soleyman et al., 2020).

By investigating the proteomes of various developmental stages of *T. canis*, researchers can identify key proteins involved in immune modulation, enzymatic activity, and host-parasite interactions (Zhu et al., 2015). Previously, 582 proteins were discovered in larval extract and 64 in ESPs of *T. canis* larvae (da Silva et al., 2018). C-type lectin TES-32, collectin-12, 26 kDa secreted antigen, heat shock protein, actin, troponin, exonuclease, and cathepsin were among the proteins potentially involved in parasite pathogenicity and evasion mechanisms (da Silva et al., 2018). A recent study also identified ESPs in *T. canis* larvae using LC-MS/MS, identifying only 19 proteins including Collectin-12, TES-26, and others (Sperotto et al., 2017). However, the protein composition of adult-stage *T. canis* ESPs, which reside long term in the intestine, remains unknown. Here, for the first time, we use MS proteomics to profile and characterize mixed-sex adult *T. canis* ESPs, advancing our understanding of its biology, virulence factors, and ability to survive in the host.

## 2 Materials and methods

### 2.1 Worm collection

More than 100 *T. canis* adult worms, including an approximately equal number of male and female worms, were collected from the intestine of a stray dog euthanized in an animal shelter in Washington DC. After being washed with phosphate buffered saline (PBS) three times, the live adult worms were cultured in 100 mL RPMI-1640 containing 100 U/mL ampicillin and 100 μg/mL streptomycin without serum in two separate 150 mm culture dishes at 37°C, 5% CO_2_ overnight.

### 2.2 ESP collection and quantification of protein concentration

The overnight culture described in section 2.1 was collected and filtered through a 0.45 µm membrane to remove debris and eggs. The culture supernatant containing *T. canis* ESPs was stored at -80°C for further use. To concentrate the ESPs of *T. canis*, 5 mL of the collected ESP sample was first transferred into a 3 kDa molecular weight cutoff (MWCO) centrifugal filter tube, as illustrated in **Figure 1**. The sample was then centrifuged at 4°C for 50 minutes at 5,000×g to concentrate the proteins. After filtration, the concentrated ESP protein solution was collected from the bottom of the filter tube, as previously described (Zhan et al., 2002). Protein concentration was determined using a Bradford assay, with a standard curve prepared using known concentrations of bovine serum albumin (BSA). A 10 µL aliquot of the concentrated ESP protein sample was mixed with 200 µL of Bradford reagent in a cuvette. The absorbance was measured at 595 nm using a spectrophotometer. The protein concentration of the ESP sample was calculated by comparing the absorbance values to the standard curve.

**Figure 1:**
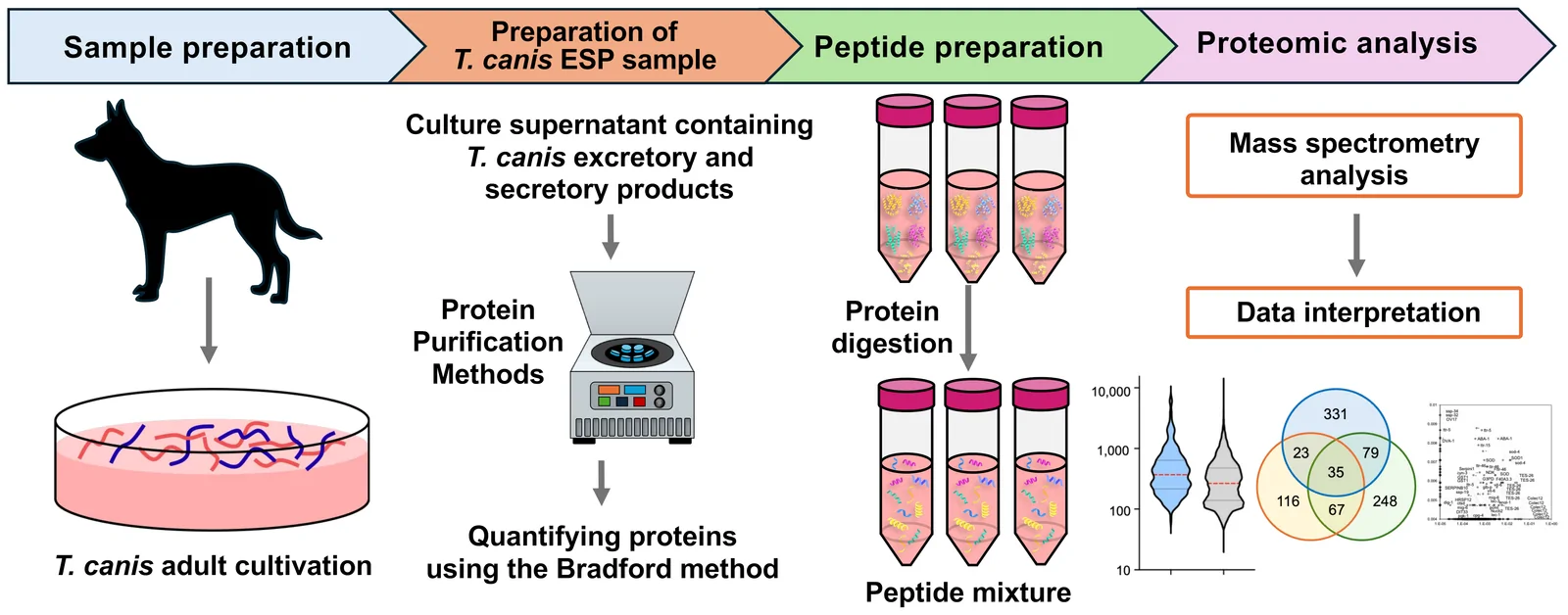
Overview of the proteomic analysis workflow. Excretory and secretory products (ESPs) were collected from mixed-sex adult *T. canis* recovered from an infected dog. The ESP samples were concentrated by centrifugal ultrafiltration to increase total protein yield, and protein content in the culture medium was quantified using the Bradford assay. The ESPs were then digested, and the resulting peptide mixtures were analyzed by LC–MS/MS. Protein identification was performed using a 1% FDR threshold. Comparative analyses, including Venn diagram visualization and cross-species abundance comparison, were used to characterize ES proteins and identify shared orthologs. Functional annotation was conducted using Gene Ontology, InterPro domain and KEGG pathway enrichment analyses to characterize the biological roles and metabolic pathways associated with ESPs.

### 2.3 Mass spectrometry

The ESP sample was purified by acetone/trichloroacetic acid precipitation. After pelleting and washing with ice-cold acetone, the resulting protein pellet was resuspended in 8M urea and 0.4 M ammonium bicarbonate, reduced with 4 mM dithiothreitol, and alkylated with 18 mM iodoacetamide. The solution was then diluted to < 2 M urea and 1 µg of trypsin was added prior to an overnight incubation at 37 °C. The resulting peptides were desalted using solid-phase extraction on the C18 spin column and the eluates were dried under a SpeedVac vacuum concentrator. The peptides were reconstituted in 0.1% formic acid and loaded onto a Neo trap cartridge coupled with an analytical column (75 µm ID x 50 cm PepMap™ Neo C18, 2 µm) over 120 min linear gradient from 2% to 35% of solvent B (0.1% formic acid in ACN) using a Vanquish Neo UHPLC System coupled to an Orbitrap Eclipse Tribrid Mass Spectrometer with FAIMS Pro Duo interface (Thermo Fisher Scientific). Data were searched using Mascot (v.3.1 Matrix Science) against the *T. canis* protein database (Zhu et al., 2015) (PRJNA248777 WBPS19) (Howe et al., 2017) and a database of universal contaminants as described in Frankenfield et al., 2022 (Frankenfield et al., 2022). Trypsin was selected as the enzyme, and the maximum number of missed cleavages was set to 3. The precursor mass tolerance was set to 10 ppm, and the fragment mass tolerance was set to 0.6 Da for the MS2 spectra. Carbamidomethylated cysteine was set as a static modification, and dynamic modifications were set as oxidized methionine, deamidated asparagine/glutamine, and protein N-term acetylation. The raw proteomics data file was uploaded to the iProX repository (Chen et al., 2022), accession #IPX0018099000.

### 2.4 Scaffold protein identification and quantification

Scaffold (version Scaffold_5.3.3, Proteome Software Inc., Portland, OR) was used to validate MS/MS based peptide and protein identifications. Peptide and protein identifications were accepted at an FDR of less than 1.0% by the Peptide Prophet algorithm (Keller et al., 2002) with Scaffold delta-mass correction, requiring at least 2 peptides per protein. Protein probabilities were assigned by the Protein Prophet algorithm (Nesvizhskii et al., 2003). Proteins that contained similar peptides and could not be differentiated based on MS/MS analysis alone were grouped to satisfy the principles of parsimony. The Scaffold sf3-format file is accessible on the iProX repository (Chen et al., 2022), accession #IPX0018099000, and the exported peptide list with spectral counts is provided in **Supplementary Table S1**. Relative protein abundance was quantified by normalized spectral abundance factor values (NSAF) (Zybailov et al., 2006), representing the relative proportion of spectral counts normalized to protein lengths (aa).

### 2.5 *T. canis* protein annotation database

For every protein in the current *T. canis* annotation (Zhu et al., 2015) (PRJNA248777 WBPS19 (Bolt et al., 2018)), gene names, gene descriptions, Gene Ontology (GO) (Gene Ontology, 2026) annotations, InterPro (Blum et al., 2025) domain annotations, BLAST matches, detection of signal peptides with SignalP (Teufel et al., 2022) and detection of transmembrane domains with TMHMM (Krogh et al., 2001) were collected from WormBase ParaSite (Howe et al., 2017). KEGG functional annotations were assigned using BlastKOALA (Kanehisa et al., 2016). The top-hit BLAST (Altschul et al., 1990) ortholog for each *T. canis* protein (E value ≤ 10^-5^) was determined using NCBI blastp (v2.7.1+) using each protein from the annotation of the *A. ceylanicum* protein set previously described in (Uzoechi et al., 2023), and to each protein from *Ascaris suum* (Jex et al., 2011) (PRJNA80881.WBPS17 (Howe et al., 2017)) and *C. elegans* (PRJNA13758, WS290 (Davis et al., 2022)). For each BLAST match, the top significant hit was considered to be the closest ortholog, but due to the large number of paralogs within each species for many of the highly detected ESPs, “reciprocal” best BLAST hits were not required for a BLAST match to be included in the analysis (i.e., the top hit in the other species may have a more significant E value to another *T. canis* protein, when the BLAST search is ran in the opposite direction). In **Supplementary Table S2**, for every *T. canis* protein, BLAST results are provided that include the top hit in the other species, the E value, the % identity (proportion of amino acids matching), the alignment length (the proportion of the protein length matching) and an indication of whether each hit was reciprocal. This BLAST data was used to align ESP detection data collected from previous studies for *A. ceylanicum* (Uzoechi et al., 2023) and for *A. suum* (Chehayeb et al., 2014). OrthoFinder v2.5.4 (Emms et al., 2026) was used to define orthologous protein families (OPFs) containing protein members spanning *T. canis*, *A. ceylanicum*, *A. suum* and *C. elegans*, using the same genome annotation versions used in BLAST searches, providing gene IDs for all orthologs and paralogs, and gene counts per OPF. The complete *T. canis* protein annotation database used for this study is available in **Supplementary Table S2**.

### 2.6 Functional enrichment analysis

GO enrichment for molecular function and cellular component terms was performed using ShinyGO v.0.82 (https://bioinformatics.sdstate.edu/go/), using the complete protein set as a background. InterPro and KEGG pathway enrichment analysis were performed on the WebGestalt (Elizarraras et al., 2024) website (version 2024) (https://www.webgestalt.org) using annotation data collected as described above. For all enrichment analyses, FDR-adjusted *P* values ≤ 0.05 were required for significance.

## 3 Results

We conducted a comprehensive LC-MS/MS-based proteomic analysis of adult *T. canis* ESPs, using ESPs collected and purified from female and male worms (**Figure 1**). Using the *T. canis* complete deduced proteome (Zhu et al., 2015) (PRJNA248777 WBPS19 (Howe et al., 2017)) as a reference database for MS analysis, we identified a total of 2,546 peptides (**Supplementary Table S1**) matched to 468 ESP proteins, at a 1% false discovery rate (FDR; see Methods), ranging in size from 62 to 8,647 amino acids. Comprehensive functional annotations were generated for all *T. canis* proteins, including KEGG (Kanehisa et al., 2025), InterPro (Blum et al., 2025) and Gene Ontology (GO) (Gene Ontology, 2026) annotations, and BLAST (Altschul et al., 1990) searches against other nematode species of interest (see Methods). The functional annotations for all proteins are provided in **Supplementary Table S2**.

The top 40 most abundant detected ESPs are shown in **Table 1**, which includes relative abundance values, functional annotations and detection of BLAST orthologs (E value ≤ 10^-5^) in proteomics ESP samples from two other nematode species: the phylogenetically closely-related Clade III (International Helminth Genomes Consortium, 2019) large roundworm *A. suum* (Chehayeb et al., 2014) (orthologs identified for 77.0% of all proteins) and the Clade V human hookworm *A. ceylanicum* (Uzoechi et al., 2023) (orthologs identified for 60.1% of all proteins). The most abundant detected ESP was a C-type lectin protein (representing 5.2% of detected protein, quantified by normalized spectral abundance factor values; NSAF (Zybailov et al., 2006)), but many of the most abundant ESPs were 26 kDa secreted antigen proteins (TES-26; n = 7 of the top 15; with many of the most abundant orthologs also being detected in *A. suum* and *A. ceylanicum* ESPs) and Collectin-12 proteins (n = 6 of the top 10), which are also C-type lectin proteins. On average, detected ESPs were longer than other *T. canis* proteins (**Figure 2A**; 593.6 aa vs. 379.2 aa; Welch’s *t*-test *P* value = 9.3×10^-24^; Cohen’s *d* effect size = 0.471). Detailed functional and conservation-based analyses of subsets of the 468 *T. canis* ESPs are provided in the sections below, to provide additional insights based on their putative involvement in parasite survival, host adaptation and pathogenicity.

**Table 1:** The top 40 most abundant ESPs of the 468 detected by MS-based proteomics in mixed-sex adult *T. canis* ESPs (1% FDR for both proteins and peptides, minimum 2 peptides).

Gene	Proteomics abundance (1% FDR)			Gene name	Gene description	*A. suum* (Chehayeb et al., 2014)	*A. ceylanicum* (Uzoechi et al., 2023)	
	# peptides	# PSMs	NSAF			Adult female NSAF	Female NSAF	Male NSAF
Tcan_01075	3	181	0.053		C-type lectin domain-containing	–	–	–
Tcan_19003	7	241	0.052	TES-26	26 kDa secreted antigen	–	–	–
Tcan_19002	7	256	0.049	TES-26	26 kDa secreted antigen	–	–	–
Tcan_00020	7	323	0.046	Colec12	Collectin-12	0	–	–
Tcan_00023	7	310	0.043	Colec12	Collectin-12	0	0	0
Tcan_00022	7	291	0.040	TES-32*	Collectin-12	0	0	0
Tcan_04685	7	179	0.034	Colec12	Collectin-12	0	0	0
Tcan_00009	7	241	0.033	TES-32/CTL-1**	Collectin-12	0	0	0
Tcan_12073	12	214	0.028	TES-26	26 kDa secreted antigen	0.0059	0.00058	0.00084
Tcan_04679	4	169	0.026	Colec12	Collectin-12	0	0	0
Tcan_00003	4	170	0.024	TES-26	26 kDa secreted antigen	–	–	–
Tcan_12049	12	180	0.024	TES-26	26 kDa secreted antigen	0.0059	0.00058	0.00084
Tcan_12345	11	168	0.023	TES-26*	26 kDa secreted antigen	0	–	–
Tcan_15190	7	66	0.022	TES-120*	26 kDa secreted antigen	–	–	–
Tcan_02129	11	68	0.015	TES-26*	26 kDa secreted antigen	–	0.00058	0.00084
Tcan_00027	15	70	0.015	sod-4	Extracellular superoxide dismutase	0.0071	0	0
Tcan_06530	16	90	0.014	SOD1	Superoxide dismutase [Cu-Zn]	0.0071	0	0
Tcan_12637	7	120	0.012	ACAN	Aggrecan core protein	0	0	0.00019
Tcan_03886	9	366	0.011	Colec12	Collectin-12	0	–	–
Tcan_02573	23	133	0.011	ASP	Ancylostoma secreted protein	0	0	0
Tcan_10200	9	56	0.010	F32A5.4	F32A5.4	–	–	–
Tcan_04905	12	73	0.010	ASP	Ancylostoma secreted protein	0	0	0
Tcan_09977	16	50	0.007	act-2b	Actin-2	0	0	0
Tcan_08064	7	90	0.007	nas-8	Superoxide dismutase [Cu-Zn]	0	0	0
Tcan_12063	11	89	0.007	TES-26	26 kDa secreted antigen	0.0059	0.00058	0.00084
Tcan_15449	12	52	0.007	TES-26	26 kDa secreted antigen	0.0059	0.00058	0.00084
Tcan_05443	7	39	0.007	sod-4	Superoxide dismutase [Cu-Zn]	0.0071	0	0
Tcan_07151	8	24	0.006		Pepsin-I3 domain-containing protein	0.0047	–	–
Tcan_06574	9	45	0.006	TES-26	26 kDa secreted antigen	0.0059	0.00058	0.00084
Tcan_13529	7	74	0.006	TES-26	26 kDa secreted antigen	0.0059	0.00058	0.00084
Tcan_13734	10	33	0.006		Pepsin-I3 domain-containing protein	–	–	–
Tcan_06369	4	43	0.005	CTL-4**	Collectin-12	0	–	–
Tcan_14077	5	16	0.005		ShKT domain-containing protein	0	–	–
Tcan_03789	24	57	0.005		ShKT domain-containing protein	–	–	–
Tcan_15720	14	34	0.005	tpi-1	Triosephosphate isomerase	0	0.00015	0.00018
Tcan_03041	10	33	0.005		ShKT domain-containing protein	0	–	–
Tcan_10301	16	58	0.004	CHIA1	Endochitinase 1	0	0	0
Tcan_17108	15	47	0.004		Actin-like protein	0.0047	0	0
Tcan_02084	6	14	0.004	ACAN	Aggrecan core protein	0	–	–
Tcan_00006	10	14	0.004	ABA-1	Polyprotein ABA-1	0.0082	0.00246	0.00589

For cross-species comparison with *A. suum* and *A. ceylanicum*, “-” = no ortholog, 0 = ortholog but no detection in ESP. NSAF = normalized spectral abundance factor values, representing normalized relative protein abundance. *T. canis larval ESPs previously detected in Sperotto et al., 2017. **T. canis larval ESP previously detected in Loukas et al., 1999.

**Figure 2:**
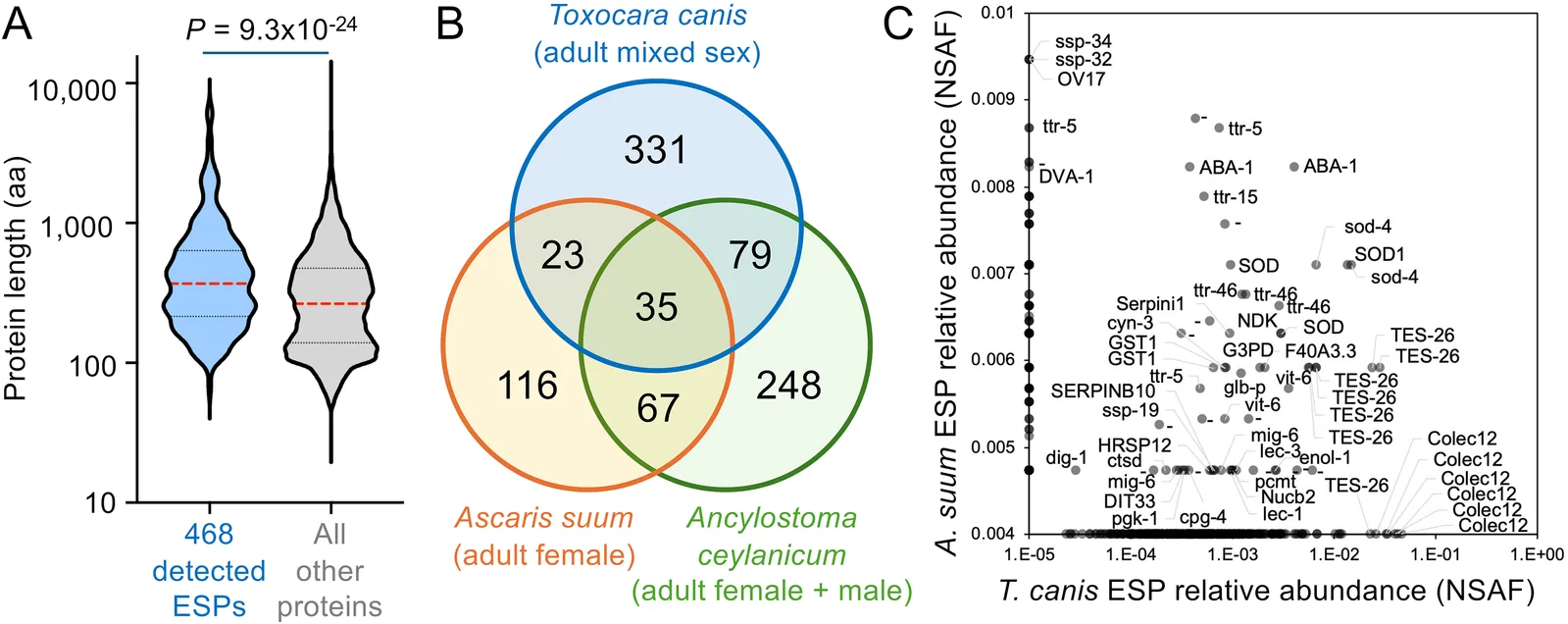
Analysis of detected ESPs from adult mixed-sex *T. canis*. **(A)** Detected ESPs are, on average, significantly longer than other proteins (Welch’s t-test, Cohen’s d effect size = 0.47). **(B)** Venn diagram comparing the proteins identified in the total adult mixed sex *T. canis* ESPs and the ESPs of adult female *A. suum* and adult female + male *A. ceylanicum*. Orthologs of *T. canis* proteins were identified in *A. suum* and *A. ceylanicum* with BLAST, and overlapping ESPs were determined based on identifications from previously published results. Across all proteins, 77.0% of *T. canis* proteins have orthologs in *A. suum* and 60.1% have orthologs in *A. ceylanicum*. **(C)** Scatterplot showing the relative protein abundance (NSAF) of ESPs from *T. canis* compared to *A. suum*. The plot illustrates the correlation in the protein abundance profiles between the two nematode species, identifying shared and species-specific protein abundance patterns in their respective ESP proteomes.

### 3.1 The 468 detected adult *T. canis* ESPs are enriched for functions including catalysis and exosome pathway

To characterize the functional properties of the *T. canis* ESPs, we performed functional enrichment analysis relative to the complete deduced protein set using GO annotations with the “molecular function” (MF) and “cellular component” (CC) parent categories (using ShinyGO (Ge et al., 2020)), as well as KEGG and InterPro annotations (using WebGestalt (Elizarraras et al., 2024)). Among the 468 identified proteins, 85 GO MF terms were significantly enriched, revealing diverse molecular activities potentially associated with host-parasite interactions and parasite survival (**Table 2**; **Supplementary Table S3**). The most significantly enriched term was Catalytic activity (GO:0003824; FDR-adjusted *P* = 8.7×10^-18^), which was annotated to 43.2% of ESPs, and many of the most significantly enriched terms were related to peptidases, oxidoreductases and binding, including the second-most enriched term, Carbohydrate binding (GO:0030246; *P_adj_*= 1.0×10^-11^, 5.3% of ESPs). Carbohydrate-binding proteins (CBPs), also known as lectins, play a crucial role in parasite-host interactions, such as facilitating host tissue recognition and invasion (Donskow-Lysoniewska et al., 2021). GO CC analysis identified significant enrichment of ESPs to the extracellular region or extracellular space, as expected (**Table 2**, complete list in **Supplementary Table S4**), in addition to enrichment detected for proteins binding to Supramolecular polymers (GO:0099081; *P_adj_*= 1.9×10^-11^) and the Extracellular matrix (GO:0031012; *P_adj_*= 2.0×10^-11^).

**Table 2:** The top 20 most significantly enriched Gene Ontology molecular function and cellular component terms for the 468 proteins detected in adult mixed-sex *T. canis* ES protein. .

Gene ontology (GO) terms			Total term size	Number of detected proteins	FDR-adjusted *P* value for enrichment (*P_adj_*)
Parent	Term	Description			
Molecular Function	GO:0003824	Catalytic activity	4455	202	8.7E-18
	GO:0030246	Carbohydrate binding	148	25	1.0E-11
	GO:0016853	Isomerase activity	116	20	1.7E-09
	GO:0016491	Oxidoreductase activity	580	43	3.1E-08
	GO:0005198	Structural molecule activity	485	37	1.8E-07
	GO:0016209	Antioxidant activity	49	12	1.8E-07
	GO:0043167	Ion binding	3316	134	2.9E-07
	GO:0016798	Hydrolase activity, acting on glycosyl bonds	107	15	4.5E-06
	GO:0004784	Superoxide dismutase activity	11	6	4.9E-06
	GO:0016721	Oxidoreductase activity, on superoxide radicals	11	6	4.9E-06
	GO:0030414	Peptidase inhibitor activity	75	12	1.5E-05
	GO:0051082	Unfolded protein binding	76	12	1.6E-05
	GO:0008233	Peptidase activity	405	29	1.8E-05
	GO:0016860	Intramolecular oxidoreductase activity	30	8	1.9E-05
	GO:0004866	Endopeptidase inhibitor activity	68	11	3.1E-05
	GO:0003779	Actin binding	211	19	4.6E-05
	GO:0061134	Peptidase regulator activity	86	12	4.6E-05
	GO:0061135	Endopeptidase regulator activity	72	11	4.6E-05
	GO:0005507	Copper ion binding	17	6	6.0E-05
	GO:0043169	Cation binding	1904	80	7.2E-05
Cellular Component	GO:0005576	Extracellular region	625	83	3.2E-34
	GO:0005615	Extracellular space	311	36	3.4E-12
	GO:0099081	Supramolecular polymer	430	41	1.9E-11
	GO:0099512	Supramolecular fiber	428	41	1.9E-11
	GO:0031012	Extracellular matrix	109	21	2.0E-11
	GO:0062023	Collagen-containing extracellular matrix	71	17	7.3E-11
	GO:0030016	Myofibril	174	25	8.2E-11
	GO:0043292	Contractile fiber	240	28	5.8E-10
	GO:0099080	Supramolecular complex	548	42	5.1E-09
	GO:0030017	Sarcomere	150	21	5.7E-09
	GO:0005829	Cytosol	889	55	1.9E-08
	GO:0005604	Basement membrane	47	12	3.3E-08
	GO:0005739	Mitochondrion	840	51	1.3E-07
	GO:0015629	Actin cytoskeleton	187	21	2.4E-07
	GO:0005856	Cytoskeleton	663	43	2.9E-07
	GO:0005759	Mitochondrial matrix	174	18	8.0E-06
	GO:0036379	Myofilament	30	8	9.6E-06
	GO:0005865	Striated muscle thin filament	23	7	1.7E-05
	GO:0031672	A band	60	10	3.6E-05
	GO:0043228	Non-membrane-bounded organelle	1689	72	8.9E-05

KEGG pathway enrichment analysis was also performed, identifying the enrichment of pathways involved in immune regulation and cell signaling among the ESPs (**Table 3**), suggesting crucial roles in modulating host–parasite interactions to facilitate long-term survival within the host. The top enriched pathways were exosome, citrate cycle (TCA cycle) and glycolysis/gluconeogenesis pathway (*P_adj_*= 7.5×10^-19^, 6.0×10^-6^ and 1.9×10^-5^, respectively). ESPs assigned to the exosome KEGG pathway among the top 40 most abundant ESPs (**Table 1**) include the cytoskeletal actins Tcan_09977 (Actin-2) and Tcan_17108 (Actin-like protein), which function as structural matrix components of extracellular vesicles (Buck et al., 2014; Wu et al., 2024), and Tcan_15720 (triosephosphate isomerase), a cytosolic glycolytic enzyme routinely exported as non-classical EV cargo (Eichenberger et al., 2018; Wu et al., 2024). As expected, ESPs were significantly enriched for signal peptides for secretion (26.5% of ESPs vs 9.9% for all proteins; *P* ≤ 10^-10^, binomial distribution test), with an additional 62 proteins being assigned to the Exosome KEGG pathway but having no signal peptides for secretion, suggesting a nonclassical exosome-facilitated secretion mechanism.

**Table 3:** KEGG pathway enrichment analysis (WebGestalt) for the 468 proteins detected in adult mixed-sex *T. canis* ES proteins, showing all 26 enriched terms.

KEGG pathway		Total term size	Number of detected proteins	FDR-adjusted *P* value for enrichment (*P_adj_*)
ID	Description			
4147	Exosome	399	69	7.45E-19
20	Citrate cycle (TCA cycle)	39	13	6.03E-06
10	Glycolysis/Gluconeogenesis	51	14	1.86E-05
1002	Peptidases and inhibitors	180	26	1.03E-04
710	Carbon fixation in photosynthetic organisms	25	9	1.68E-04
3110	Chaperones and folding catalysts	113	19	2.21E-04
5020	Prion diseases	18	7	8.90E-04
630	Glyoxylate and dicarboxylate metabolism	39	10	8.90E-04
5016	Huntington disease	119	18	1.23E-03
30	Pentose phosphate pathway	27	8	1.62E-03
5146	Amoebiasis	22	7	2.75E-03
5134	Legionellosis	16	6	2.97E-03
4213	Longevity regulating pathway - multiple species	34	8	7.47E-03
620	Pyruvate metabolism	45	9	1.06E-02
4964	Proximal tubule bicarbonate reclamation	28	7	1.07E-02
5014	Amyotrophic lateral sclerosis (ALS)	37	8	1.09E-02
720	Carbon fixation pathways in prokaryotes	21	6	1.09E-02
4142	Lysosome	67	11	1.09E-02
4940	Type I diabetes mellitus	4	3	1.09E-02
4812	Cytoskeleton proteins	170	19	1.96E-02
4151	PI3K-Akt signaling pathway	76	11	2.87E-02
4141	Protein processing in endoplasmic reticulum	112	14	2.87E-02
680	Methane metabolism	19	5	3.83E-02
4512	ECM-receptor interaction	6	3	3.93E-02
4146	Peroxisome	69	10	3.93E-02
4217	Necroptosis	48	8	4.17E-02

Functional enrichment of proteins based on InterPro (Blum et al., 2025) (IPR) domains was also performed, identifying 58 significantly enriched domains (**Table 4**; **Supplementary Table S5**). The top enriched domains included *S. helianthus* K-toxin (ShKT; *P_adj_*= 7.2×10^-16^) and several PEBP domains (which are both associated with TES-26 proteins), as well as C-type lectin domains and peptidases.

**Table 4:** The top 20 most significantly enriched InterPro domains (WebGestalt) among the 468 proteins detected in adult mixed-sex *T. canis* ES proteins.

InterPro domain		Total term size	Number of detected proteins	FDR-adjusted *P* value for enrichment (*P_adj_*)
Term	Description			
IPR003582	ShKT domain	85	28	7.2E-16
IPR035810	Phosphatidylethanolamine-binding protein eukaryotic	15	11	2.1E-10
IPR036610	PEBP-like superfamily	15	11	2.1E-10
IPR008914	Phosphatidylethanolamine-binding protein	12	9	1.8E-08
IPR038412	Pepsin inhibitor-3 domain superfamily	39	14	3.5E-08
IPR036249	Thioredoxin-like superfamily	93	20	9.9E-08
IPR010480	Pepsin inhibitor-3-like repeated domain	33	12	4.9E-07
IPR001858	Phosphatidylethanolamine-binding conserved site	7	6	5.6E-06
IPR013766	Thioredoxin domain	35	11	1.1E-05
IPR001304	C-type lectin-like	63	14	1.9E-05
IPR016187	C-type lectin fold	85	16	2.4E-05
IPR040079	Glutathione transferase family	11	6	2.2E-04
IPR036282	Glutathione S-transferase C-terminal domain superfamily	27	8	8.7E-04
IPR001506	Peptidase M12A	38	9	1.6E-03
IPR034035	Astacin-like metallopeptidase domain	31	8	2.3E-03
IPR004046	Glutathione S-transferase C-terminal	16	6	2.4E-03
IPR000034	Laminin IV	6	4	3.7E-03
IPR027410	TCP-1-like chaperonin intermediate domain superfamily	11	5	3.7E-03
IPR001283	Cysteine-rich secretory protein-related	18	6	4.4E-03
IPR027409	GroEL-like apical domain superfamily	12	5	4.5E-03

### 3.2 Orthology based comparisons identify conserved and *T. canis*-specific ESPs and related functions

Next, we performed a comparative proteomic analysis to identify phylogenetically conserved and unique proteins between *T. canis*, the closely related roundworm *A. suum* (adult female stage) and the human hookworm *A. ceylanicum* (adult male and female, combined for analysis; **Figure 2B**). We identified that 331 of the 468 detected *T. canis* ESPs (70.7%) were uniquely detected in *T. canis*, compared to 116 (48.3%) in *A. suum*, and 248 (57.8%) in *A. ceylanicum*. The 35 ESPs that were shared between the three nematodes (**Table 5**) include six TES-26 proteins, six transthyretin proteins and ABA-1, previously identified as a major allergen in both *T. canis* and *A. suum* (Kennedy et al., 1995). An additional 23 *T. canis* ESPs had detected ESP orthologs only in *A. suum*, with the most abundant of the 58 total overlapping ESPs including two ABA-1 orthologs, and several superoxide dismutase (SOD) proteins (**Figure 2C**). Overall, most ESPs were only detected in one species, with only a small portion being conserved, reflecting critical roles in rapid adaptation for host interactions and parasite survival in their species-specific niches and host tropism. The 331 *T. canis*-specific ESPs (full list in **Supplementary Table S6**) include the top 8 most abundant detected overall ESPs, with similar enrichment to the overall 468 detected ESPs.

**Table 5:** The 35 proteins detected by MS Proteomics in all the *T. canis* mixed-sex adult-stage ESPs sample, *A. suum* female adult ESPs and *A. ceylanicum* adult female + male ESPs. .

Gene	Proteomics abundance (1% FDR)			Gene name	Gene description
	# peptides	# PSMs	NSAF		
Tcan_04307	2	3	0.000736	ttr-5	Transthyretin-like protein 5
Tcan_04457	2	3	0.000670	ssp-19	Major sperm protein
Tcan_02177	2	2	0.000645	SERPINB10	Serpin B10
Tcan_15427	2	2	0.000601	–	Major sperm protein isoform beta
Tcan_17206	2	3	0.000594	HRSP12	Ribonuclease
Tcan_07555	2	2	0.000524	ttr-15	Transthyretin-like protein 15
Tcan_09248	2	2	0.000481	ttr-5	Transthyretin-like protein 5
Tcan_00015	2	2	0.000383	ABA-1	Polyprotein ABA-1
Tcan_12657	2	2	0.000327	DIT33	Pepsin inhibitor Dit33
Tcan_12307	3	4	0.000299	–	Cell surface protein
Tcan_06386	4	6	0.001230	ttr-46	Transthyretin-like protein 46
Tcan_05477	4	6	0.000860	cyn-3	Peptidyl-prolyl cis-trans isomerase
Tcan_06372	5	10	0.002880	ttr-46	Transthyretin-like protein 46
Tcan_08355	5	11	0.002083	F40A3.3	Phosphatidylethanolamine-binding-like protein F40A3.3
Tcan_18218	5	6	0.000339	pgk-1	Phosphoglycerate kinase
Tcan_11021	5	5	0.000029	dig-1	Mesocentin
Tcan_06354	6	9	0.001363	ttr-46	Transthyretin-like protein 46
Tcan_14042	6	8	0.000958	lec-1	Galectin
Tcan_13529	7	74	0.005629	TES-26	26 kDa secreted antigen
Tcan_06677	7	18	0.003030	NDK	Nucleoside diphosphate kinase
Tcan_11644	7	10	0.001010	pcmt	Protein-L-isoaspartate(D-aspartate) O-methyltransferase
Tcan_17563	8	22	0.001888	G3PD	Glyceraldehyde-3-phosphate dehydrogenase
Tcan_06574	9	45	0.005786	TES-26	26 kDa secreted antigen
Tcan_05396	9	14	0.003030	SOD	Superoxide dismutase [Cu-Zn]
Tcan_14145	9	13	0.001002	Nucb2	Nucleobindin-2
Tcan_00006	10	14	0.004031	ABA-1	Polyprotein ABA-1
Tcan_12063	11	89	0.006697	TES-26	26 kDa secreted antigen
Tcan_06489	11	15	0.001084	lec-3	Galectin
Tcan_10482	11	28	0.000929	Serpini1	Neuroserpin
Tcan_12073	12	214	0.028459	TES-26	26 kDa secreted antigen
Tcan_12049	12	180	0.023937	TES-26	26 kDa secreted antigen
Tcan_15449	12	52	0.006686	TES-26	26 kDa secreted antigen
Tcan_04454	18	33	0.002637	enol-1	phosphopyruvate hydratase
Tcan_05025	26	41	0.000844	vit-6	Vitellogenin-6
Tcan_12573	88	210	0.003601	vit-6	Vitellogenin-6

## 4 Discussion

Understanding the mechanisms by which *T. canis* interacts with the host may offer insights into immunity evasion and offer potential novel targets for treatment and prevention. In this study, a proteomic analysis of *T. canis* ESPs was performed, providing an overview of the secreted protein composition and biological functions. The combined database of results provided in **Supplementary Table S2** provides a convenient and accessible resource for exploring functional annotations, ESP abundance and orthology across species, for all *T. canis* proteins.

As shown in **Table 1**, many *T. canis* 26 kDa secreted antigen (TES-26) paralogs were among the most abundant detected proteins. TES-26 proteins are phosphatidylethanolamine-binding proteins with two ShK/SXC domains, and maintain a hydrophobic motif that facilitates lipid binding; the combination of TES-26 and ShK/SXC motifs suggests an additional role functioning as a highly immunogenic antigen and serving as a molecular effector that manipulates host physiology (Długosz et al., 2021). TES-26 proteins were previously identified as key antigens for diagnosis and potential vaccine development (Długosz et al., 2021; Varghese et al., 2017), and two highly detected paralogs were detected among only 19 previously-described *T. canis* larval ESPs (Sperotto et al., 2017) (Tcan_12345 and Tcan_02129, both in the top 15 most abundant proteins), with expression detection in a previous larval study (Gems et al., 1995). We also detected a high abundance of a previously described monosaccharide-binding TES-26 ortholog (Loukas et al., 1999) (r-CTL; Tcan_00009, the 8^th^ most abundant ESP) which has also been expressed recombinantly and improved disability scores in mice induced with autoimmune encephalomyelitis (Shahbakhsh et al., 2025), and TES-70/CTL-4 (Tcan_06369, 32^nd^-most abundant), previously identified as a larval ESP that binds to host glycans, potentially downregulating host immune responses (Loukas et al., 2000). Mammalian C-type lectins are involved in various biological processes, notably pathogen recognition, functioning as pattern recognition receptors (PRRs) (van den Berg et al., 2012). They recognize carbohydrate structures on a wide range of pathogens, such as bacteria, viruses, fungi, and parasites, often in a Ca^2+^-dependent manner (van den Berg et al., 2012), and previous studies have reported the secretion of C-type lectins during the larval (L2) stage (Hewitson et al., 2009).

We detected six Collectin-12 C-type lectins among our top 10 most abundant adult-stage *T. canis* ESPs, the orthologs of which were not detected in *A. suum* or *A. ceylanicum* ESPs (**Table 1**, **Figure 2B**). Additionally, two of our highly-detected CTL-like ESPs (TES-32 or TES-120) show structural similarity to mammalian lectins, and play key roles in the activation of innate immunity by binding to carbohydrates, including monosaccharides and glycoconjugates (Shahbakhsh et al., 2025). Overall, the high relative abundance of many TES and C-type lectin proteins in the *T. canis* adult ESPs indicates their apparent importance in host-parasite interactions. The number of distinct paralogs also highlights the complexity of understanding each of their specific roles and interactions, which warrants future study.

Several superoxide dismutase (SOD) ESPs were highly abundant in both *T. canis* and *A. suum*. SOD, its intracellular isoform SOD1, and GST (also detected in both species) are typically induced in response to host immune challenge, and may contribute to defense against host-derived oxidative stress and facilitate the detoxification of reactive oxygen species (ROS), supporting parasite survival (Xu et al., 2020). In the filarial nematode *Onchocerca volvulus*, a SOD protein in ESPs was found to be the target of human humoral immune activation, inducing strong IgG responses (Ajonina-Ekoti et al., 2012). Two ABA-1 orthologs were also highly abundant in the ESPs of both species, and these have been previously identified as a major allergen in both *T. canis* and *A. suum (*Kennedy et al., 1995*)*. Additionally, transthyretin-like proteins (ttr-5 and ttr-46) are highly abundant in both *T. canis* and *A. suum*, representing a conserved nematode-specific family involved in lipid-binding, developmental signaling, and environmental sensing (Hennebry et al., 2006).

Overall, the 468 ESPs were dominated by TES-26 proteins (14 total ESPs, 24.6% of total detected protein), Collectin-12 C-type lectins (8 ESPs, 19.8% of total detected protein), and SOD/GST proteins (12 ESPs, 4.9% of detected proteins).

Among the KEGG pathways enriched in *T. canis* ESPs (**Table 3**), the exosome pathway was the most significantly enriched. Nematode extracellular vesicles (EVs) including exosomes have been described for their role in the delivery of microRNAs, proteins, lipids, and nucleic acids capable of modulating host immune functions by suppressing Type 2 innate immune responses (Buck et al., 2014), including EVs from the hookworm *Nippostrongylus brasiliensis* which can prevent inducible colitis in mice (Eichenberger et al., 2018). The identification of 69 exosome-associated proteins among the 468 *T. canis* adult ESPs likely represents an important mechanism for the delivery of *T. canis* ESPs to host tissues.

Antigenic cross-reactivity with other parasitic nematodes is a recognized limitation of *Toxocara* serodiagnosis, particularly with *Ascaris* spp. in areas where ascariasis is endemic (Fan and Su, 2004; Muñoz-Guzmán et al., 2010; Nunes et al., 1997). *A. suum*, the large roundworm of pigs, is the sibling species of the human roundworm *A. lumbricoides*. The two species are so closely related morphologically and genetically that their status as separate species remains debated, and cross-infection occurs between humans and pigs, with worms reaching the adult stage in the small intestine of either host (Leles et al., 2012; Nejsum et al., 2005). Although ELISA or Western blot remains a widely used diagnostic method in many laboratories, it often relies on antigens that may lack sufficient specificity. Therefore, the development of highly specific antigens is essential to minimize cross-reactivity and reduce the risk of misdiagnosis or inappropriate treatment. In a previous examination, blood samples from patients suspected of VLM were analyzed using western blot, revealing a molecular weight (MW) of around 55–66 kDa, which appears to be in part responsible for the cross-reactivity between *T. canis* and *A. suum* (Nunes et al., 1997). Among our detected ESPs, 58 were found to be orthologous between *T. canis* and *A. suum* (**Figure 2B**), including a galectin protein (lec-3, Tcan_06489) with a MW of 54.1 kDa and a phosphoglycerate kinase (pgk-1, Tcan_18218) with a MW of 66.2 kDa, which are the two shared ESPs with the closest MW to the previously identified cross-reactive antigen (Nunes et al., 1997).

Considering both BLAST matches and OrthoFinder-derived orthologous protein families (OPFs; see Methods), the top four most abundant *T. canis*-specific proteins with no orthology to *A. suum* or *A. ceylanicum* were all TES-26 orthologs belonging to the same OPF (OG0000712). Together, these four comprise 14.8% of the total ESP proteome (Tcan_19003, 5.2%; Tcan_19002, 4.9%; Tcan_00003, 2.4%; Tcan_15190, 2.2%). Tcan_15190 was also one of the 19 previously described *T. canis* larval ESPs (labeled as TES-120 in (Sperotto et al., 2017)), which also suggests that this may be an optimal target for diagnosis, with high detection consistently across life cycle stages, and high specificity to *T. canis*.

## 5 Conclusion

This study provides the first proteomic profile of adult-stage *T. canis* ESPs, offering new insights into the mechanisms underlying parasite survival, migration, and host interaction. The resulting proteomic resource identifies potential diagnostic biomarkers for toxocariasis and highlights species-specific ESPs that may contribute to host–pathogen interactions. In addition, orthology analyses provide insight into the conservation and specificity of key ESP targets, while the identification of proteins involved in immune modulation and tissue invasion advances our understanding of *T. canis* biology. Collectively, these findings establish a foundation for future studies in comparative parasitology and support the development of improved diagnostics, vaccine candidates, and other targeted control strategies for toxocariasis.

## Data Availability

The original contributions presented in the study are publicly available. This data can be found here: https://www.iprox.cn//page/project.html?id=IPX0018099000.

## Supplementary material

Supplementary Table 1 Peptide sequences and spectral counts. Data is from Scaffold v5.3.3, requiring FDR ≤ 1% for peptides and proteins, and a minimum of 2 peptides per protein.

Supplementary Table 2 Proteomics detection statistics, functional annotations, and orthology data for all Toxocara canis proteins.

Supplementary Table 3 Gene Ontology molecular function enrichment (ShinyGO) for the 468 proteins detected in adult mixed-sex Toxocara canis ESP.

Supplementary Table 4 Gene Ontology cellular component enrichment (ShinyGO) for the 468 proteins detected in adult mixed-sex Toxocara canis ESP.

Supplementary Table 5 InterPro domain enrichment (WebGestalt) for the 468 proteins detected in adult mixed-sex Toxocara canis ESP.

Supplementary Table 6 The 331 species-specific ESPs detected from Toxocara canis mixed-sex adults.
